# Lysozyme and Its Application as Antibacterial Agent in Food Industry

**DOI:** 10.3390/molecules27196305

**Published:** 2022-09-24

**Authors:** Nida Nawaz, Sai Wen, Fenghuan Wang, Shiza Nawaz, Junaid Raza, Maryam Iftikhar, Muhammad Usman

**Affiliations:** 1 School of Light Industry, Beijing Technology and Business University (BTBU), Beijing 100048, China; 2 Constituent College of Pakistan Institute of Engineering and Applied Sciences (PIEAS), Faisalabad 38000, Pakistan; 3 Institute of Biotechnology, Brandenburg University of Technology (BTU) Cottbus-Senftenberg, 01968 Senftenberg, Germany; 4 Laboratory of Molecular Sensory Science, Beijing Technology and Business University (BTBU), Beijing 100048, China; 5 Beijing Advanced Innovation Center for Food Nutrition and Human Health, China-Canada Joint Lab of Food Nutrition and Health Beijing, Beijing Technology and Business University (BTBU), Beijing 100048, China

**Keywords:** lysozyme, catalytic effect, non-catalytic effect, bacterial resistance, modification, natural food preservative

## Abstract

Lysozymes are hydrolytic enzymes characterized by their ability to cleave the β-(1,4)-glycosidic bonds in peptidoglycan, a major structural component of the bacterial cell wall. This hydrolysis action compromises the integrity of the cell wall, causing the lysis of bacteria. For more than 80 years, its role of antibacterial defense in animals has been renowned, and it is also used as a preservative in foods and pharmaceuticals. In order to improve the antimicrobial efficacy of lysozyme, extensive research has been intended for its modifications. This manuscript reviews the natural antibiotic compound lysozyme with reference to its catalytic and non-catalytic mode of antibacterial action, lysozyme types, susceptibility and resistance of bacteria, modification of lysozyme molecules, and its applications in the food industry.

## 1. Introduction

The increasing demand for healthy food products has led to reforms in quality and safety control of the food industry. Now, natural antimicrobial compounds are gaining interest in the food industry as food preservatives with reduced demand for chemical additives. These natural antimicrobials are produced and isolated from different sources, including plants, animals, and microorganisms. These compounds are used to extend the shelf life of food products by killing or inhibiting microbial growth, and they are trending among customers [1].

Lysozyme (EC.3.2.1.17) is an antimicrobial protein widely distributed in many biological tissues, cells, and body fluids [2]. It belongs to a class of glycoside hydrolase that can hydrolyze the carbohydrate chains in bacterial cell walls, which is an important mesh-like saccule that encloses the cell, conferring shape and strength against osmotic pressure. The lysozyme-targeted component of the bacterial cell wall is peptidoglycan (PG), which is composed of glycan chains of alternating *N*-acetylglucosamine (NAG) and *N*-acetylmuramic acid (NAM) that are cross-linked by peptides associated with the lactyl moiety of NAM. Lysozyme, also called 1,4-β-d-*N*-acetyl muramidase, hydrolyzes the glycosidic bond between the first carbon of NAM and the fourth carbon of NAG, and hence elicits the disintegration of the bacterial cell. Thus, lysozyme exhibits strong antibacterial properties against bacteria and is practically applicable in food and pharmaceutical industries. Lysozyme is most abundant in the egg white and can also be readily found in secretions, including tears, saliva, human milk, and mucus. Till now, diverse lysozymes have been identified in animals, plants, microbes, as well as in some viruses. Some typical sources of lysozymes, together with their amount are shown in Table 1 adapted from [3].

Lysozyme is an important part of the innate immune system and exhibits strong antimicrobial activities against bacterial, fungal, and viral pathogens. It protects against infections, acts as a natural antibiotic, and enhances the efficacy of other antibiotics, while it also strengthens the immune system [4]. In pharmaceutical industries, lysozyme can be applied for the prevention of many diseases of bacterial, viral, fungal, and inflammatory origins and also exerts immune stimulatory and antihistaminic effects [5,6]. The improved lysozyme even provides new opportunities in the field of clinical medicine. The protein was suggested to conduce to the destruction of tumors, as it modulates the synthesis of the tumor necrosis factor (TNFα) and also stimulates the production of Type I interferon (INFα, INFβ, INFγ), interleukin-2 (II-2) and interleukin-6 (IL-6) by human lymphocytes [7]. In the current pandemic of the coronavirus, some modified form of lysozyme can be used to stimulate the formation of interferon, an effective substance against coronavirus, and thus reduce the risk of the life-threatening form of COVID-19 up to 79% [8,9]. 

Lysozyme is more suitable for food systems with their potential applications to be directly added to or coated on the surface of food to prevent the growth of detrimental microbes. These proteins, along with other antimicrobials, have also found potential applications as food preservatives [10]. This manuscript reviews this natural antimicrobial compound with reference to its applications, particularly in food systems. 

This review collected and summarized the articles from “Web of Science Core Collection” database in Web of Science with keywords of “lysozyme” and subtitles in this paper. Several chapters of books relevant to the topic were also included. The research area was mainly restrained to “Food Science Technology”. Most of the uncovered papers were published from early 2000 till now.

## 2. Types of Lysozymes

Despite the consensus on functionality, there are different types of lysozymes differentiated by their amino acid sequence, structure, physicochemical and immunological properties. Lysozymes have conventionally been classified into three categories, including c-type (chicken-type or conventional-type), g-type (goose-type), and i-type (invertebrate-type) lysozyme.

C-type lysozyme is a model protein for the study of enzymology and structural biology. It is renowned for its hydrolysis of the (1-4) glycosidic bond in the cell wall of Gram-positive bacteria, a property that has already been widely applied in food protection [11]. C-type lysozyme is found in all vertebrates, and it can also be found in different classes of *Phylum Arthropoda,* mainly in insect species of *lepidopteran* [12], *dipteran* [13], *isopteran* [14], and *hemipteran* [15]. In this big family of lysozyme, the chicken egg white lysozyme (HEWL) and human lysozyme are classic representatives. Human lysozyme is the first sequenced mammalian lysozyme. Human lysozyme, along with HEWL, has been widely used over the past 30 years as a model system to study the protein structure, function, and particularly the mechanism of protein folding and protein stability [16]. For food or pharmaceutical use, human lysozyme has the edge over HEWL for being low immunogenic and hypoallergenic. However, the use of human lysozyme is largely restricted because of its limited source. So far, recombinant human lysozyme has been expressed in plants [17], animals [18], bacteria [19], and yeasts [20].

G-type lysozyme was first identified in the egg whites of the Embden goose and hence named as g-type. It is the major type of lysozyme in the bird species, such as rhea [21] and cassowary [22]. Functional g-type genes have been identified in invertebrates, such as some mollusks [23] and urochordates [24]. 

I-type lysozyme is the third type of lysozyme found in the animal kingdom. As indicated by its name, i-type lysozyme is mainly found in invertebrates. It is confirmed that i-type lysozyme occurs in the phyla of annelids, echinoderms, nematodes, and arthropods [25]. Some i-type lysozymes have shown significant antibacterial activity against Gram-negative bacteria. Xue et al. (2007) reported two i-type lysozymes in the eastern oyster that significantly inhibited the growth of *Escherichia coli, Vibrio vulnificus,* and *Pediococcus cerevisiae* [26]. A special i-type lysozyme, the destabilase-lysozyme from medicinal leech, was discovered to be a multifunctional enzyme with isopeptidase and glycosidase activities [27]. Identification of different types of lysozymes (c-type, g-type, i-type) in the animal kingdom has been summarized (Table 2). 

## 3. Mode of Action of Lysozyme

Lysozyme efficiency can be challenged in its ability to control bacterial growth. The generally recognized mechanism adapted by this protein is the enzymatic degradation of the glycosidic β-linkage in the cell wall to kill the sensitive bacteria. However, increasing evidence suggests that lysozyme has additional bactericidal mechanisms towards bacteria beyond those related to the catalytic action. 

### 3.1. Catalytic Mode of Antibacterial Action

**Figure 1:**
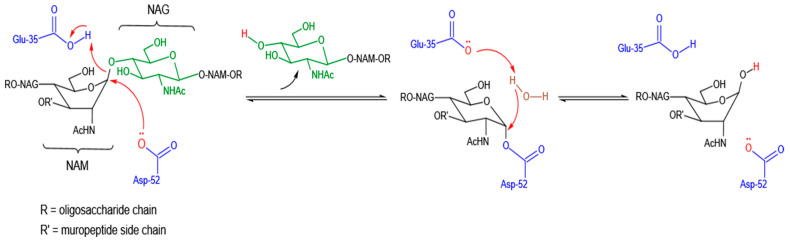
Hydrolytic mechanism of action of lysozyme on β(1–4) linkages between NAM and NAG residues of the bacterial cell wall backbone.

Lysozyme functions by attacking, hydrolyzing, and breaking the muco polysaccharide part of the PG in the bacterial cell wall. Similarly, this enzyme can also break glycosidic bonds in chitin. The lysozyme molecule generally employs a compact, globular structure with the hydrophilic groups of residues exposed on the surface and hydrophobic ones clustering internally. To accommodate the long chain substrate, there is a deep groove on the surface of lysozyme. This groove is the active site involved in binding to the bacterial carbohydrate chain and subsequently cleaving it. The binding substrate is a polysaccharide of six amino sugars long and is positioned along the active site by hydrogen bonding and hydrophobic interactions [11]. During this fitting process, the strain on the glycosidic bond between 4th and 5th sugar unit increases, and thus the carbon-oxygen bond between them will be broken by a general-acid catalyst residue, glutamic acid (Glu) and a general-base catalyst residue, aspartic acid (Asp) or cysteine (Cys) in the active site of lysozyme. In this reaction, glutamic acid acts as a proton donor through the free carbonyl group of its side chain, whereas aspartic acid acts as a nucleophile to produce a glycosyl-enzyme intermediate. This intermediate product immediately reacts with a water molecule and generates the hydrolysis product (Figure 1) [35]. Besides the Glu and Asp residues, a third catalytically important residue—threonine (Thr) or serine (Ser), serving as a catalytic water positioning residue (in the sequence of Glu-8aa-Asp/Cys-5aa-Thr catalytic triad), was previously demonstrated for lysozymes of coliphages T4 and P21 [36]. As an exception, goose egg-white lysozyme (GEWL) has only a single catalytic residue-Glu, suggesting that a second acidic residue is not essential for the catalytic activity of goose lysozyme [37]. This phenomenon has also been observed in a lytic transglycosylase, i.e., phage lambda endolysin [38] and an endolysin of *Burkholderia* AP3 phage with lysozyme-like catalytic subunit [39].

The distinct catalytic characteristics and spatial structures of diverse lysozymes determine their ability to break down the bacterial skeleton. There are two distinct lysozymes discovered in *Enterococcus hirae* ATCC 9790 (also named as *Streptococcu faecium*). The first one, muramidase-1, is proved to be a glucoenzyme and is multiply nucleotidylated with an unusual nucleotide, 5-mercaptouridine monophosphate. Moreover, muramidase-1 is a zymogen and requires protease activation [40]. The extracellular muramidase-2, on the other hand, possesses several unusual features. It appears to consist of two or perhaps three functional modules that are linked in a single polypeptide chain. The glycosidase-active site locates in the N-terminal domain, while the six 45-amino-acid-long repeats at the C-terminal domain are suggested to be involved in binding to the PG substrate with high affinity [41]. Interestingly, muramidase-2 was also shown to bind penicillin G with low affinity in the presence of a typical SXXK motif and other amino acid motifs that are characteristic of penicillin-interactive proteins, which form the putative third domain [42]. Such modular architecture as also been discovered in lysozymes from the lytic *Streptococcus pneumoniae* bacteriophages, i.e., Cpl-1 and Cpl-7. These two phage lysozymes contain a virtually identical N-terminal catalytic module (85.6% identical and 90.9% similar), but differ in their C-terminal binding module, which encompasses repeated sequences. The repeat-unit lengths are 20 amino acids (aa) and repeat six times in Cpl-1, contributing to its attachment to the choline moieties of pneumococcal (lipo) teichoic acids [43]. By contrast, there are three identical repetitions of 42 amino acids (CW_7 repeats) found in Cpl-7, which endows this lysozyme with a specific activity of hydrolyzing choline- as well as ethanolamine-containing pneumococcal cell walls [44,45]. 

Afore chitinase, other polysaccharide-degrading enzymes, such as endolysins of some lytic bacteriophages [46], cellulase [47], chitinase [48], xylanase [49], and alginate lyases [50] were also characterized by the modular organization. This observation gave us a hint that such modular construction with separate catalytic and substrate-binding domains may be advantageous to the enzymes that deal with bulky and insoluble polysaccharide substrates.

It has been observed that the enzymatic microbicidal activity of lysozyme is generally restricted to Gram-positive bacteria. This phenomenon is mostly due to the presence of lipopolysaccharides (LPS), lipoproteins, and some hydrophobic peptides in the outer membrane of Gram-negative bacteria, which hinders the access of lysozyme to the nether layer of PG [51]. Besides LPS barrier, certain modifications of PG layer have also been proved to prevent the effective binding of lysozyme, such as *N*-deacetylation of NAG, *O*-acetylation of NAM, and *N*-glycosylation of NAM, which is further discussed in this review. These observations reflect the ongoing arms race between lysozyme and targeted microorganism in the long period of evolution. Some distinct antimicrobial mechanism of lysozyme other than catalytic action has been uncovered as well in the past decades.

### 3.2. Non-Catalytic Mode of Antibacterial Action

**Figure 2:**
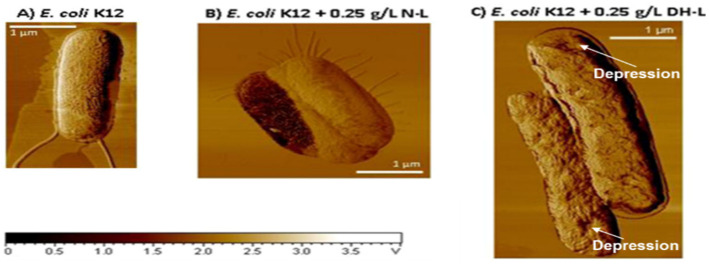
AFM phase imaging: Non-treated *E. coli* K12 cells (**A**); cells treated with native lysozyme (N-L) at 0.25 g/L (**B**); cells treated with dry-heated lysozyme (DH-L) at 0.25 g/L (**C**). The z-range is from 0 to 4 V. “Reprinted with permission from [52]. Copyright © 2022 American Chemical Society”.

Researchers had long believed that lysozyme only exerts its antimicrobial activity by functioning as a hydrolytic enzyme. However, there is emerging evidence showing that non-enzymatic microbicidal activity rather than hydrolytic activity of lysozyme contributes substantially to the killing of microbes. A direct proof is that lysozyme with less or no enzymatic activity had maintained, or even improved, bactericidal activity against both Gram-positive and Gram-negative bacteria. It was shown that heat denaturation of HEWL, resulting in an enzymatically inactive, more cationic, and hydrophobic dimeric form, enhanced the bactericidal activity against Gram-negative bacteria (Figure 2) [52].

**Figure 3:**
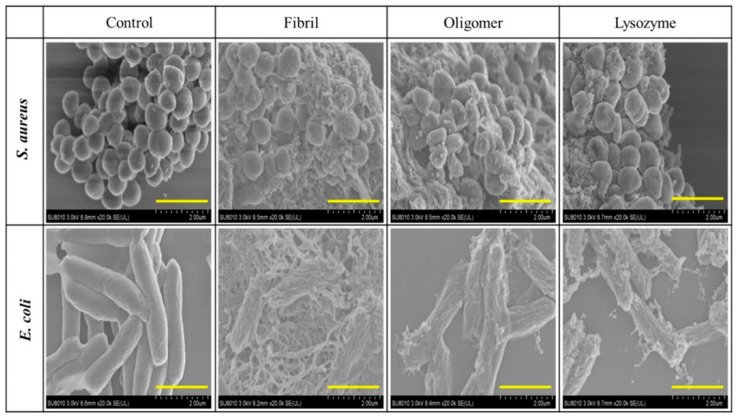
SEM images of *S. aureus* and *E. coli* under the exposure of buffer (Tris−HCl, pH 7.2, control), HEWL fibril, HEWL oligomer, and HEWL after 6 h. Scale bar, 2 μm. “Reprinted with permission from [53]. Copyright © 2022 American Chemical Society”.

Two recent studies on the amyloid fibrils of HEWL and its amyloid-like aggregates have both demonstrated significantly enhanced antibacterial activity of self-assembled HEWL against lysozyme-resistant *Staphylococcus aureus* and lysozyme-insensitive *E. coli* comparing with native HEWL [53,54] (Figure 3). Notably, the amyloid fibrils were not constructed by full-length HEWL. The core structure of the fibrils was mainly composed of the peptides 49–101 and 53–101 of HEWL, which were produced by heat (90 °C) and acidic treatment (pH = 2) with abundant β-sheet conformation. These studies have revealed that lysozyme possesses both enzymatic and non-enzymatic antibacterial activities.

It is suggested that the cationic, hydrophilic, and lipophilic nature of lysozyme contributes to its attraction to the negatively charged outer membrane of bacteria and disruption of the integrity of the plasma membrane [55]. This concept was further supported by the discoveries of innate antimicrobial peptides (AMPs) in lysozymes of different types, such as HEWL [56], T4 phage lysozyme [57], GEWL (g-type) [58], and also i-type lysozyme-liked destabilase-lysozyme (DL) [59]. Unlike the lysozyme protein, lysozyme-derived peptides exhibited in vitro antimicrobial activities against both Gram-negative and Gram-positive bacteria. For instance, the helix-loop-helix (HLH) peptides located at the upper lip of the active site cleft of HEWL (residues 87–114) and human lysozyme (residues 87–115) were both synthesized and proved to confer potent antimicrobial activity with membrane permeabilization efficacy [60]. These peptides mostly reside at the terminal region(s) of lysozyme and are surface-exposed. The α-helical and amphiphilic features are highly conserved in these peptides, which are in accord with the membrane-binding amphipathic helix (AH), a motif commonly found in natural AMPs. It has been demonstrated that, owing to the amphiphilicity of helical peptide, the polar residues of one face likely interact with the polar lipid head groups, and the hydrophobic ones of the opposite face drive membrane binding through hydrophobic effect [61]. Several studies on the lipid-binding properties of lysozyme have suggested that the membrane association of lysozyme is driven by both electrostatic and hydrophobic interactions [62]. 

Besides binding to the membrane, bactericidal efficacy of lysozyme probably also requires cell penetration and perturbations of the membrane. A general model of cationic AMPs suggests that, they can displace the divalent cations and bind to the LPS due to their greater affinity for the LPS. In this way, AMPs disrupt the salt bridges between phosphate groups and divalent cations and cause transient cracks permitting passage of the peptide itself across the membrane [63]. When approaching the cytoplasmic membrane, these peptides accumulate to a critical concentration and induce significant perturbations and disorganizations of the lipid membrane, resulting in loss of the transmembrane potential and additional membrane dysfunction, such as inhibition of ATP production and proton motive force, eventually leading to cell death [64]. However, studies on the action mode of AMPs only provide molecular clues to the behavior of lysozyme, things are more complicated when considering that the innate AMPs of lysozyme are restrained in the protein skeleton and may work in coordination with other residues in the protein. Therefore, the dynamic processes and molecular mechanisms of lysozyme against bacteria have not been deciphered completely and still present a challenge. Nevertheless, discoveries of the non-catalytic cation mode, especially the innate antibacterial peptide in lysozyme, open up new opportunities for mutagenesis of lysozyme by protein engineering strategy for a more potent antimicrobial agent [65].

## 4. Susceptibility and Resistance of Bacteria to Lysozyme

Gram-negative bacteria consist of an outer membrane and a middle membrane with a single layer of rigid PG lodged in the periplasmic proteins. The outer membrane is made up of LPS, lipoproteins, and phospholipids [66]. The structure of LPS is different from that of phospholipids in that it contains multiple hydrophobic gel-like lipid chains covalently linked to a large, negatively charged polysaccharide. This unique structure leads to a gel state of very low fluidity at the center of the outer membrane that blocks polar solutes, whereas the hydrated core region, with its strong charge interactions, impedes the movement of hydrophobic molecules [67,68]. Therefore, the resistance of Gram-negative bacteria to many substances, such as antibacterial peptides, some hydrophobic molecules, and antibiotics (e.g., penicillin), is mainly due to the barrier of the outer membrane [69,70]. The LPS and its lipid A portion (endotoxic unit) were found to bind with lysozyme by electrostatic interaction between lysozyme and the lipid A-phosphates [71]. 

The introduction of high-resolution analytical techniques and genetic approaches shed light on the biological functions of the cell wall and other determinants of naturally lysozyme-resistant bacteria [72,73]. The resistance mechanisms have been extensively studied in both Gram-positive and Gram-negative bacteria, though the mechanisms depend on the species.

### 4.1. Modifications of Peptidoglycan

The orthodox mechanism for bacterial killing by lysozyme occurs through the hydrolysis of cell wall PG. There are several PG-related mechanisms suggested for the resistance in Gram-positive and Gram-negative bacteria. Three types of PG modifications have been mostly observed in lysozyme-resistant bacteria to prevent the effective killing by lysozyme. These are *N*-deacetylation, *O*-acetylation, and *N*-glycolylation of the sugar moieties, which are limited to the -NH_2_ group at C2 and the -OH group at C6 of the sugars [74].

#### 4.1.1. *N*-Deacetylation of NAG or NAM

**Figure 4:**
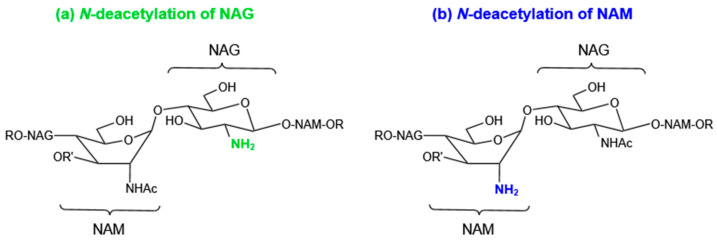
*N*-deacetylation of NAG (**a**) or NAM (**b**).

The efficient hydrolytic activity has been assisted by the interactions between the active site of lysozyme and the acetyl groups on the glycan backbone of PG. To lessen such type of interactions, many pathogenic bacteria can express a NAG deacetylase to remove the acetyl group at the C2 position of NAG (Figure 4). This deacetylase was encoded by pgdA. *Streptococcus pneumonia* is more sensitive to lysozyme due to the absence of pgdA [75]. Some pathogenic bacteria including *Enterococcus faecalis*, *Helicobacter pylori*, *Listeria monocytogenes*, *Streptococcus suis*, *Streptococcus iniae*(*pdi*), *Mycobacterium tuberculosis* (*Rv1096*), and *Clostridium difficile* (*pdaV*) have pgdA homologs and thus enhanced bacterial resistance to lysozyme [76]. NAG deacetylation is mostly reported in Gram-positive bacteria, with only a few exceptions, e.g., Gram-negative bacteria *Shigella flexneri* [77]. 

In *Bacillus subtilis*, two polysaccharide deacetylase homologs, PdaA and PdaC can catalyze the removal of the acetyl group from the NAM. The enzyme PdaA is implicated in the δ-lactam formation of *B. subtilis* spore cell wall, while its homologs are also encoded in the genomes of non-spore-forming microorganisms, e.g., *Rhizobium leguminosarum*. Researchers hypothesized that PdaA activity is important for bacteria to evade the innate immune system because the absence of acetyl group in NAM interferes with the binding of muramyl dipeptide (MDP) to the NOD2 receptor and the activation of the subsequent signaling cascade [78]. 

#### 4.1.2. *O*-Acetylation of NAM

**Figure 5:**
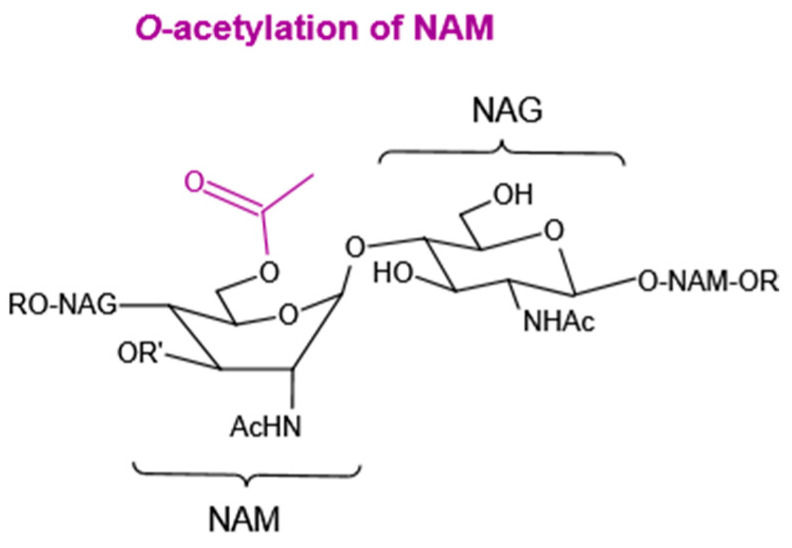
*O*-acetylation of NAM.

*O*-acetylation is defined as the addition of an acetyl group to the C6 hydroxyl group of NAM. It is a common modification observed in many Gram-positive and Gram-negative bacteria, but with different mechanisms [79]. It prevents the binding of lysozyme to the PG due to steric hindrance caused by the bulky acetyl group (Figure 5) [80]. Contrary to NAM *O*-acetylation, NAG *O*-acetylation is very infrequent in bacteria.

In Gram-positive bacterium *S. aureus*, its resistance to lysozyme is enhanced by the *O*-acetyltransferase A (OatA), as O acetylates the NAM of PG [81]. On the other hand, loss of NAM O-acetyltransferase activity due to mutagenesis in *L. monocytogenes* (*oatA*), *S. pneumoniae* (*adr*), and *Bacillus anthracis* (*oatB*) resulted in enhanced sensitivity towards lysozyme [82]. 

In Gram-negative bacteria, both *patA* (or *pacA*) and *patB* (or *pacB*) gene products are required for the *O*-acetylation of NAM. *PatA* (*or PacA*) is a transmembrane protein related to the transfer of acetate from the cytoplasm to the periplasm, and *PatB* (*or PacB*) is the periplasmic *O*-acetyltransferase [79]. *Neisseria gonorrhoeae* and *Neisseria meningitides* harboring the *pacA* and *pacB* genes are resistant to lysozyme [83]. In recent research, it was proved that *pacA* does not affect the sensitivity of *N. gonorrhoeae* to lysozyme except when the bacterial envelope integrity is also considered [72].

#### 4.1.3. *N*-Glycolylation of NAM

**Figure 6:**
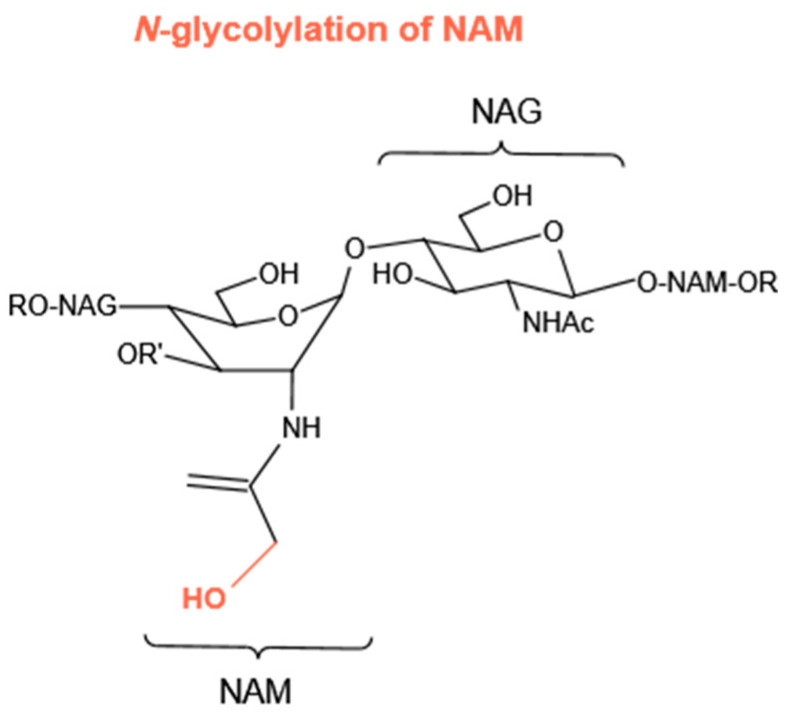
*N*-glycolylation of NAM.

Compared with the *N*-deacetylation of NAG and *O*-acetylation of NAM, there are few bacterial species that *N*-glycolylate their PG (Figure 6). This kind of modification in the PG is only observed in *Mycobacteria* and five other closely related genera of bacteria. In *Mycobacteria,* a mono-oxygenase enzyme (hydroxylase), encoded by the gene *namH,* is responsible for the production of *N*-glycolylmuramic acid. Therefore, it was reported that *Mycobacterium smegmatis* has decreased resistance to lysozyme due to the loss of *namH* [84].

### 4.2. Specific Proteinaceous Inhibitors of Lysozyme

In recent years, specific proteinaceous lysozyme-inhibitors have been incorporated into the mechanisms of improved resistance to lysozyme. Several Gram-negative bacteria can protect themselves against the enzymatic activity of host lysozymes by producing periplasmic proteins [85]. So far, four different families of lysozyme inhibitors have been identified, including Ivy (Inhibitor of vertebrate lysozyme), MliC/PliC (Membrane-associated/periplasmic inhibitor of c-type lysozyme), PliI and PliG (periplasmic inhibitors of i- and g-type lysozymes, respectively). Besides these, a novel secretory lysozyme inhibitor has been discovered in virulent *Streptococcus pyogenes* strains (M1 and M57). It was named the streptococcal inhibitor of complement (SIC) [86]. These inhibitors are the first of their kind, and their crystal structures, or in complex with their cognate lysozyme, have unraveled their mode of interaction with lysozyme [87]. Taking Ivy_Ec_ (PDB 1XS0), for example, a rigid loop protrudes into the HEWL active-site cleft in a key-lock type of interaction, and the central histidine in this loop connects with the two catalytic residues (D52 and E35) via hydrogen bonds. Based on the deciphered interaction mechanism and critical residues, it has been demonstrated possible to genetically engineer lysozymes to evade pathogen-derived inhibitory proteins via gene mutagenesis and an innovative ultrahigh-throughput screening platform [88].

### 4.3. Modifications of Anionic Glycopolymers of Cell Wall

Wall teichoic acids (TWAs) and their attached substituents, the unique components of Gram-positive bacterial cell wall, can affect bacterial cell surface charge and hydrophobicity, thus impeding the binding of extracellular molecules. In *S. aureus*, a teichoic acid can be covalently attached to the C6 hydroxyl group of NAM and contributes to an increased resistance to lysozyme through steric hindrance [85]. By preventing *D*-alanylation through gene deletion of *dlt* operon, which removed D-alanine esters from teichoic acids, the *S. aureus* mutant lacking D-alanine has an increased susceptibility to lysozyme [89]. 

In Gram-negative bacteria, which are devoid of teichoic acids, LPS is a central factor implicated in the cell permeability and antibiotic resistance. Two deep rough mutants of *Salmonella Typhimurium* LT2 with truncated LPS in their outer membrane had shown spheroplast formation in the presence of lysozyme (1000 µg/mL), but without the aid of EDTA, which is a prerequisite for spheroplast formation of parent strain [90]. It has been reported that *Salmonella enterica* have evolved a defense mechanism, PhoPQ two-component regulatory system, which triggers the modification of lipid A portion of LPS for an increased resistance to host cationic AMPs [91]. In fact, such LPS (lipid A) modifications were demonstrated to help to stabilize the outer membrane of *S. enterica* by strengthening the lateral interactions between neighboring LPS molecules and the divalent cation bridging network and thus prevent the penetration of large molecules like AMPs [4]. However, the degree to which this affects Gram-negative resistance to lysozyme is largely unknown. Another example is *Acinetobacter baumannii* mutant, in which phosphor ethanolamine was added to the lipid A portion of lipopolysaccharide by enhancing the activation of PmrAB2 component signal transduction system, resulting in a reduced negative charge of the cell wall and increased resistance to the lysozyme [92]. 

## 5. Modification of Lysozyme

The bacterial membrane is disrupted specifically by molecules that are positively charged and amphipathic in nature. Lysozymes, at their physiological pH, also have these physiochemical. However, there is a need for modifications in lysozyme as its activity against lysozyme-insensitive bacteria is limited. These modified lysozymes can depict new antimicrobial properties against bacteria to a greater extent [52].

In protein chemistry, some chemical modifications are used in molecules to manufacture derivative products with novel functions. These chemical modifications may be of different types, such as acetylation, phosphorylation, glycosylation, esterification, and succinylation [93]. The glycosylation can modify the net charge of the protein surface, so the protein-protein and protein-water interactions were affected [94]. Consequently, the isoelectric point and conformation of protein changed, and interfacial behavior of glycosylated proteins can be analyzed [94,95]. During the last two decades, many chemical and enzymatic methods have been developed to improve the efficacy of lysozyme [96,97]. Two different types of alterations were reviewed in this section. 

### 5.1. Lipophilization of Lysozyme

Lipophilization alters the properties of proteins through esterification using small lipophilic moieties (fatty acid or fatty alcohol). Some short and middle chain saturated fatty acids, such as myristic acid, were also attached to lysozymes to improve the bactericidal action [98]. The bactericidal activity of lysozyme increased with the number of short chain fatty acids attached to it [99]. Both glycosylation and lipophilization of lysozyme molecules could be used as a potential treatment for industrial applications [100]. The glycosylation of lysozyme produced stable proteins, and thus, they were highly resistant to protease action. These proteins have improved charge effects and water-binding capacity [101]. It was further confirmed that an egg white lysozyme first modified by glycosylation and later lipophilized by palmitic acid increased the yield of lipophilized lysozyme. This molecule also showed enhanced antimicrobial activity against *E. coli.* Thus, lipophilization combined with glycosylation is a promising method for lysozyme modification applicable to industry [102].

### 5.2. Modifications with Polysaccharides

Proteins and polysaccharides are the main components of foods that contribute to the functional properties of the food systems [103]. These are widely used in the food industry for stabilization of food emulsions products either in native or modified forms. The common modification method typically includes protein–polysaccharide conjugate to enhance the activities of lysozyme and lysozyme polysaccharide complexes. There are many factors that may affect the conjugation of lysozymes with polysaccharides. They may include different pH, temperature, and weight ratios between polysaccharide and lysozyme. Several techniques can be used for characterization of the conjugations, for example, SDS-PAGE, fast protein liquid chromatography (FPLC), Fourier transform infrared spectroscopy (FT-IR), and detection of free amino groups of lysozyme. Further, for the antimicrobial and functional studies, large-scale ion exchange and gel exclusion chromatography may also be used [104]. 

It has been indicated that different polysaccharides may conduce to unusual properties of lysozyme under similar conditions, which depends on their conformation, size, water solubility, and the reducing end of the aldehyde group. These changes in properties are mostly embodied in their behavior against different bacteria [94,105]. 

Different methods were studied and used to overcome the allergenic risks caused by the presence of lysozyme in food and beverages. A renowned Millard reaction is used for the attachment of polysaccharides to lysozyme. Millard reaction consists of a series of chemical reactions between the carbonyl group of carbohydrates and the amino group of proteins [104]. The polysaccharide-conjugated lysozymes could be considered novel biopolymers that might have great potential to act as effective natural antibacterial agents in the food systems [106] and also extend the shelf life of wrapped foods [107]. Some polysaccharides, such as dextran, galactomannan, chitosan, gum Arabic, and xanthan gum, are conjugated with lysozymes for research studies (Table 3).

## 6. Application of Lysozymes in the Food Industry

Food is the necessity of life and value-added product in modern society. However, it can become unhealthful when it undergoes chemical, physical or enzymatic changes and thus lead to massive economic loss in food industries. To address these issues, effective strategies were used to control the spoilage in food, including thermal treatment, modified packaging, water activity (a_w_) control, nutrient restriction, use of antimicrobials, etc. The use of natural antibacterial agents in food processing and food preservation has played a vital role in controlling foodborne illness and food poisoning and reduced the longstanding concerns on the impact of chemical antibacterial agents on human health [113]. 

Lysozyme is an antimicrobial protein naturally present in substantial amounts in mammalian milk and avian eggs, and hence generally recognized as safe (GRAS) for direct addition in foods. In lieu of traditional antibiotics, lysozymes can be used to preserve food and beverages. It was used in the food processing of a variety of food mainly by adding to the final product and also used as a protective matrix [106]. The early industrial application of this enzyme was its addition to the hard cheese backdated to the 1970s. The World Health Organization (WHO) also allows the use of lysozyme as a food preservative, frequently in sushi, Chinese noodles, cheese, etc. 

The major food pathogens in the food industry include *L. monocytogenes* and *C. botulinum,* both can cause severe illness and even death [114]. In ready-to-eat products where bacterial growth is possible, no *L. monocytogenes* will be allowed. Lysozyme has been proven effective for controlling *L. monocytogenes* in pork, beef, pork sausages, and turkey frankfurters [115]. Lysozyme can also control the toxin formation caused by *C. botulinum* proliferation in poultry, fish, and vegetables. *C. tyrobutyricum* is responsible for the texture deterioration and unpleasant taste in various types of ripening cheese. Lysozyme was used to inhibit the growth of *C. tyrobutyricum* in cheese production in the dairy industry [116]. 

In the beverage processing industry, lysozymes are also used to produce fermented beverages. Lysozyme does not affect yeast growth. Therefore, it can be used before or during alcoholic fermentation. These enzymes are commonly added to control the growth of Gram-positive spoilage bacteria, such as *Pediococcus* and *Lactobacilli* in wine and beer brewing, so high quality of these products can be ensured [117]. It can be a useful tool in the stabilization and prevention of unwelcomed organisms without the reliance on higher levels of sulfur dioxide. The foam stability of wines treated with charcoal can also be increased by using lysozyme and also clarifies red wines [3]. Lysozyme also has some sweetening properties and is used as a natural sweetener in the food industry [118].

Besides application as food preservatives, lysozyme has recently been used to produce a nano sensor for the detection of metal ions, such as Hg^2+^, a pollutant in food sources causing damage to the central nervous system, endocrine system, brain, and even kidney [119]. Using HEWL as reducing and stabilizing agents, researchers synthesized the gold fluorescent clusters (GFC) in basic aqueous solution, which is a selective label-free and highly sensitive sensor for Hg^2+^ though Hg^2+^ specific quenching of GFC. The detection limit of this lysozyme-stabilized GFC is as low as 10 nM. Owing to its non-toxicity and highly fluorescent property, this sensor has promising prospects in food quality control.

In recent years, intelligent packaging has become a novel solution for food quality and safety. It prolongs the quality and stability of food products without any radiation, thermal or high-pressure treatments. Antimicrobial packaging is the most challenging and interesting topic nowadays in food systems. Lysozyme is applied as a component of food packaging to extend the shelf life of various processed foods inhibiting microbial growth. These can be incorporated into film or coating upon the food surface. It may diminish the risk of pathogen contagion and expand the shelf life. A combination of one or more antimicrobials is recently used for food packaging [120]. These antimicrobials can be directly added or coated on the surface of food to prevent the growth of detrimental microbes. 

Bisphenol S (BPS), an endocrine-disrupting compound extensively used in food packaging products, causes severe health hazards. In a recent study, an interaction between lysozyme and BPS was revealed by multi-spectroscopic and theoretical approaches. Lysozyme interacts with BPS through static quenching, whereas hydrophobic force directs the underlying interactions. Results of molecular docking revealed that tryptophan is essential in binding, thus this structural alteration of lysozyme may change its functional properties as a food preservative [121].

The recent trends in food systems include the various methods of lysozyme immobilization. The most effectual one is the immobilization of HEWL by adsorption. It especially increased the lysozyme antimicrobial properties against Gram-negative bacteria. It has been studied that chitosan-lysozyme films showed excellent antimicrobial activity against *E. coli* and *S. faecalis* [3]. In another study, it has been reported that lysozyme-conjugated nanocellulose had the best antifungal and antibacterial effects against strains of *S. aureus, Candida albicans, E. coli*, etc. [122]. 

The most common process in antimicrobial active food packaging systems is the controlled release of antimicrobial protein to maintain the safety and quality of food. For this purpose, polyvinyl alcohol (PVOH) and polyethylene terephthalate (PET) films have been used for the immobilization of lysozyme. Malhotra et al. [123] reported that lysozyme was incorporated in PVOH films to confer sustained release of antimicrobials for inhibition, and the degree of cross-linking of these PVOH films affected this release process. Moreover, the efficacy of lactoferrin-coated PET film also has been reported to reduce the population of hydrogen sulpide (H_2_S) producing bacteria, mainly including *Shewanellaputre faciens* and *Pseudomonas* [124]. When lysozyme was introduced to the film of whey proteins, it inhibited the development of *L. monocytogenes* in salmon. In the case of films based on zein, lysozymes inhibited the growth of *Lactobacillus plantarum* and *B. subtilis*. Furthermore, it was demonstrated that when lysozyme was injected into the film of zein combined with EDTA, it enhanced the effect against the Gram-negative bacteria *E. coli*. Compared with the simple and straight-forward incorporation of lysozyme into food, antimicrobial films elicit a longer period of food protection [125].

A novel and effective antimicrobial strategy developed by covalently conjugating antioxidant phenolic compound gentistic acid (GA) with antibacterial lysozyme. These dual-functional conjugates simultaneously mitigate both the lipid oxidation and microbial growth. The LYZ:GA mixing ratio controls the particle size, antioxidant activity, morphology, and antimicrobial performance of the resultant conjugates. The findings revealed that maximum antioxidant activity and antibacterial performance of the conjugates is achieved when LYZ:GA molar ratio is 1:112. Thus, these LYZ-GA conjugates potentially become more effective dual-functional ingredients to combat food waste and loss in the future [126].

Development of edible food packaging films with antimicrobial properties is required for healthy and safe food as plastic-based food packaging causes environmental pollution. Lactoperoxidase (LP) and lysozyme are the two best antibacterial enzymes for food preservation. Recently, applications of LP and LYZ-containing edible coatings with excellent functional properties were reviewed. These edible coatings were integrated with other biomolecules such as alginate, chitosan, whey protein, and gelatin, to improve food packaging. Mostly fresh food, poultry, and seafood products have LP and lysozyme-coated edible films as these are stable, safe, and strong antimicrobial properties. Therefore, the use of these edible coatings, in addition to other methods in food preservation, can achieve a synergy effect [10]. 

Recently, a nontoxic and safe green antibacterial preservation material was prepared and used for food preservation. A water-soluble *N*-succinyl chitosan (NSC) was prepared by reacting succinic anhydride with chitosan. When NSC was loaded with lysozyme, a Lysozyme-N-succinyl chitosan (LYZ-NSC) was obtained to check the effect of NSC on lysozyme activity and antibacterial activity. The results revealed that lysozyme activity in LYZ-NSC was increased by 256% as compared to free lysozyme activity, thus, increasing bacteriostatic activity at low concentrations. Both the structure and stability of NSC and LYZ-NSC were analyzed and compared by the introduction of an active group located in the C2–NH_2_ group of chitosan. The results revealed that the change in the secondary structure of lysozyme during lysozyme loading process may lead to a change in lysozyme activity. Based on these properties, both NSC and NSC-LYZ can be used in food preservation methods and specifically prolong the freshness of strawberries. NSC-LYZ effectively extended the shelf life of strawberries by three days. Thus, it provides a feasible green preservative material for storage of food [127].

## 7. Conclusions

Natural antimicrobials are attractive among food technologists, and this field is gaining interest to use as alternatives to previous chemical- and physical-based antimicrobials. Lysozymes have emerged as effective natural antimicrobials and therefore have the status of GRAS by USFDA. However, there are still many limitations regarding the consumer choice, efficacy, cost, large-scale production of these antimicrobials. Therefore, development is needed in the food industry for large-scale production of these antimicrobials from natural sources with their functional activity so that they may approve for their regular use in the future. 

Further challenges in the use of natural antimicrobials are the strong marketing strategies and involvement of some regulatory actions to decertify the chemical preservatives widely used. Thus, natural antimicrobials provide an incredible opportunity to develop the field of food production. Additionally, this review revealed that modified lysozymes are exceptionally promising natural food preservatives to be used in the food industry.

## Figures and Tables

**Table 1:** Selected sources of lysozyme.

Source of Lysozyme	Amount of Lysozyme
Tears	3000–5000 µg/mL
Chicken egg white	2500–3500 µg/mL
Duck egg white	1000–1300 µg/mL
Goose egg white	500–700 µg/mL
Human milk	55-75 µg/mL
Cow milk	10–15 µg/mL
Cauliflower juice	25–28 µg/mL
Cabbage juice	7–8 µg/mL
Papaya juice	9 µg/mL
Spleen	50–160 mg/kg
Thymus	60–80 mg/kg
Pancreas	20–35 mg/kg

**Table 2:** Identification of c-type, g-type, and i-type lysozymes in the animal kingdom.

Type of Lysozyme	Class	Organism	Type of Identification	References
**c-type**	Birds	Chicken	AA sequence	[21]
	Mammals	Human	AA sequence	[28]
	Insects	Lepidoptera	cDNA isolation	[12]
		Diptera	cDNA isolation	[13]
		Isoptera	cDNA isolation	[14]
		Hemiptera	cDNA isolation	[15]
**g-type**	Birds	Goose	AA sequence	[29]
		Cassowary	AA sequence	[22]
		Rhea	AA sequence	[21]
	Fish	Japanese flounder	cDNA isolation	[30]
		Atlantic cod	cDNA isolation	[31]
	Mammals	Human	Similarity search with chicken lysozyme in databases	[32]
	Invertebrates	Mollusks	cDNA isolation	[23]
		Urochordates	cDNA isolation	[24]
**i-type**	Mollusks	*Tapes japonica*	cDNA isolation and AA sequence	[33]
	Echinodermata	Sea cucumber	cDNA isolation	[34]

**Table 3:** Modified Lysozymes with their properties.

Modified Lysozyme	Properties	Reference
Palmitic acid	Antimicrobial activity against *E. coli* and *Edwardsiella tarda.*	[108]
Short and middle chain saturated fatty acids	Improve the bactericidal action	[99]
Dextran	Excellent in vitro antibacterial effect against *S. aureus* and *E. coli.*	[109]
Dextran	Preparation of a dextran-lysozyme conjugate for antibacterial effect against *S. aureus* and *E. coli* in a natural food system (cheese curd)	[105]
Dextran	Increased heat stability, better emulsion and higher solubility	[109]
Glactomannan	Antimicrobial activity against the Gram-negative pathogen *E. tarda*	[110]
Chitosan	Exhibits antimicrobial action towards *E. coli* K-12	[111]
Chitosan	lysozyme-chitosan composite film activated against *E. coli* and *Streptococcus faecalis*	[112]
Xanthan gum	Used as a thickener, stabilizer, and an emulsifier in the food industry. Inhibited the growth of *S. aureus* and *E. coli.*	[104]

## Abbreviations

**AH**	amphipathic helix	**MDP**	muramyl dipeptide
**AMPs**	antimicrobial peptides	**MliC**	membrane associated inhibitor of c-type lysozyme
**BPS**	bisphenol S	**NAG**	*N*-acetylglucosamine
**DL**	destabilase-lysozyme	**NAM**	*N*-acetylmuramic acid
**GEWL**	goose egg-white lysozyme	**NSC**	*N*-succinyl chitosan
**GFC**	gold fluorescent clusters	**OatA**	*O*-acetyltransferase A
**GRAS**	generally recognized as safe	**PG**	peptidoglycan
**HEWL**	hen/chicken egg white lysozyme	**PET**	polyethylene terephthalate
**HLH**	helix-loop-helix	**PliC**	periplasmic inhibitor of C-type lysozyme
**II-2**	interleukin-2	**PliI**	periplasmic inhibitors of I-type lysozyme
**IL-6**	interleukin-6	**PliG**	periplasmic inhibitors of G-type lysozymes
**Ivy**	inhibitor of vertebrate lysozyme	**PVOH**	polyvinyl alcohol
**LP**	lactoperoxidase	**SIC**	streptococcal inhibitor of complement
**LPS**	lipopolysaccharides	**TNFα**	tumor necrosis factor
**LYZ**	lysozyme	**TWAs**	wall teichoic acids
**LYZ-NSC**	lysozyme-*N*-succinyl chitosan		

## References

[1] Juneja V.K., Dwivedi H.P., Yan X. (2012). Novel natural food antimicrobials. Annu. Rev. Food Sci. Technol..

[2] Lesnierowski G., Stangierski J. (2018). What’s new in chicken egg research and technology for human health promotion?—A review. Trends Food Sci. Technol..

[3] Liburdi K., Benucci I., Esti M. (2014). Lysozyme in wine: An overview of current and future applications. Compr. Rev. Food Sci. Food Saf..

[4] Ragland S.A., Criss A.K. (2017). From bacterial killing to immune modulation: Recent insights into the functions of lysozyme. PLoS Pathog..

[5] Tagashira A., Nishi K., Matsumoto S., Sugahara T. (2018). Anti-inflammatory effect of lysozyme from hen egg white on mouse peritoneal macrophages. Cytotechnology.

[6] Khan M.I., Dowarha D., Katte R., Chou R.-H., Filipek A., Yu C. (2019). Lysozyme as the anti-proliferative agent to block the interaction between S100A6 and the RAGE V domain. PLoS ONE.

[7] Liao A.-H., Hung C.-R., Lin C.-F., Lin Y.-C., Chen H.-K. (2017). Treatment effects of lysozyme-shelled microbubbles and ultrasound in inflammatory skin disease. Sci. Rep..

[8] Shalhoub S. (2020). Interferon beta-1b for COVID-19. Lancet.

[9] Acharya D., Liu G., Gack M.U. (2020). Dysregulation of type I interferon responses in COVID-19. Nat. Rev. Immunol..

[10] Zhang W., Rhim J.W. (2022). Functional edible films/coatings integrated with lactoperoxidase and lysozyme and their application in food preservation. Food Control.

[11] Leśnierowski G., Yang T. (2021). Lysozyme and its modified forms: A critical appraisal of selected properties and potential. Trends Food Sci. Technol..

[12] Kim J.W., Yoe S.M. (2003). Analysis of a lysozyme gene from sweet potato hornworm, Agrius convolvuli. Entomol. Res..

[13] Daffre S., Kylsten P., Samakovlis C., Hultmark D. (1994). The lysozyme locus in Drosophila melanogaster: An expanded gene family adapted for expression in the digestive tract. Mol. Genet. Genom..

[14] Fujita A., Minamoto T., Shimizu I., Abe T. (2002). Molecular cloning of lysozyme-encoding cDNAs expressed in the salivary gland of a wood-feeding termite, Reticulitermes speratus. Insect Biochem. Mol. Biol..

[15] Araújo C.A., Waniek P.J., Stock P., Mayer C., Jansen A.M., Schaub G.A. (2006). Sequence characterization and expression patterns of defensin and lysozyme encoding genes from the gut of the reduviid bug Triatoma brasiliensis. Insect Biochem. Mol. Biol..

[16] Takano K., Yamagata Y., Yutani K. (2000). Role of amino acid residues at turns in the conformational stability and folding of human lysozyme. Biochemistry.

[17] Huang J., Wu L., Yalda D., Adkins Y., Kelleher S.L., Crane M., Lonnerdal B., Rodriguez R.L., Huang N. (2002). Expression of functional recombinant human lysozyme in transgenic rice cell culture. Transgenic Res..

[18] Yang B., Wang J., Tang B., Liu Y., Guo C., Yang P., Yu T., Li R., Zhao J., Zhang L. (2011). Characterization of bioactive recombinant human lysozyme expressed in milk of cloned transgenic cattle. PLoS ONE.

[19] Dan L., Liu S., Shang S., Zhang H., Zhang R., Li N. (2018). Expression of recombinant human lysozyme in bacterial artificial chromosome transgenic mice promotes the growth of Bifidobacterium and inhibits the growth of Salmonella in the intestine. J. Biotechnol..

[20] Gao Y., Zhao H.L., Feng X., Zhai R.D., Zhu S., DU C.T., Sun C.J., Lei L.C. (2013). Expression of recombinant human lysozyme-tachyplesin I (hLYZ-TP I) in Pichia pastoris and analysis of antibacterial activity. Biomed. Environ. Sci..

[21] Pooart J., Torikata T., Araki T. (2004). The primary structure of a novel goose-type lysozyme from rhea egg white. Biosci. Biotechnol. Biochem..

[22] Thammasirirak S., Torikata T., Takami K., Murata K., Araki T. (2002). The primary structure of cassowary (*Casuarius casuarius*) goose type lysozyme. Biosci. Biotechnol. Biochem..

[23] Zhao J., Song L., Li C., Zou H., Ni D., Wang W., Xu W. (2007). Molecular cloning of an invertebrate goose-type lysozyme gene from Chlamys farreri, and lytic activity of the recombinant protein. Mol. Immunol..

[24] Nilsen I.W., Myrnes B., Edvardsen R.B., Chourrout D. (2003). Urochordates carry multiple genes for goose-type lysozyme and no genes for chicken-or invertebrate-type lysozymes. Cell. Mol. Life Sci..

[25] Paskewitz S., Li B., Kajla M. (2008). Cloning and molecular characterization of two invertebrate-type lysozymes from Anopheles gambiae. Insect Mol. Biol..

[26] Xue Q.G., Itoh N., Schey K.L., Li Y.L., Cooper R.K., La Peyre J.F. (2007). A new lysozyme from the eastern oyster (*Crassostrea virginica*) indicates adaptive evolution of i-type lysozymes. Cell. Mol. Life Sci..

[27] Kurdyumov A.S., Manuvera V.A., Baskova I.P., Lazarev V.N. (2015). A comparison of the enzymatic properties of three recombinant isoforms of thrombolytic and antibacterial protein—Destabilase-Lysozyme from medicinal leech. BMC Biochem..

[28] Peters C.W., Kruse U., Pollwein R., Grzeschik K.H., Sippel A.E. (1989). The human lysozyme gene: Sequence organization and chromosomal localization. Eur. J. Biochem..

[29] Simpson R.J., Morgan F.J. (1983). Complete amino acid sequence of Embden goose (*Anser anser*) egg-white lysozyme. BBA Protein Struct. Mol. Enzymol..

[30] Hikima J., Minagawa S., Hirono I., Aoki T. (2001). Molecular cloning, expression and evolution of the Japanese flounder goose-type lysozyme gene, and the lytic activity of its recombinant protein. BBA-Gene Struct. Expr..

[31] Larsen A.N., Solstad T., Svineng G., Seppola M., Jørgensen T.Ø. (2009). Molecular characterisation of a goose-type lysozyme gene in Atlantic cod (*Gadus morhua* L.). Fish Shellfish Immunol..

[32] Irwin D.M., Gong Z.M. (2003). Molecular evolution of vertebrate goose-type lysozyme genes. J. Mol. Evol..

[33] Takeshita K., Hashimoto Y., Thujihata Y., So T., Ueda T., Iomoto T. (2004). Determination of the complete cDNA sequence, construction of expression systems, and elucidation of fibrinolytic activity for Tapes japonica lysozyme. Protein Expr. Purif..

[34] Cong L., Yang X., Wang X., Tada M., Lu M., Liu H., Zhu B. (2009). Characterization of an i-type lysozyme gene from the sea cucumber *Stichopus japonicus*, and enzymatic and nonenzymatic antimicrobial activities of its recombinant protein. J. Biosci. Bioeng..

[35] Vocadlo D.J., Davies G.J., Laine R., Withers S.G. (2001). Catalysis by hen egg-white lysozyme proceeds via a covalent intermediate. Nature.

[36] Wohlkönig A., Huet J., Looze Y., Wintjens R. (2010). Structural relationships in the lysozyme superfamily: Significant evidence for glycoside hydrolase signature motifs. PLoS ONE.

[37] Weaver L., Grütter M., Matthews B. (1995). The refined structures of goose lysozyme and its complex with a bound trisaccharide show that the “Goose-type” ly’sozymes lack a catalytic aspartate residue. J. Mol. Biol..

[38] Vollmer W., Blanot D., De Pedro M.A. (2008). Peptidoglycan structure and architecture. FEMS Microbiol. Rev..

[39] Yang M.M., Wang J., Dong L., Kong J., Teng Y., Liu P., Fan J.J., Yu X.H. (2017). Lack of association of C3 gene with uveitis: Additional insights into the genetic profile of uveitis regarding complement pathway genes. Sci. Rep..

[40] Shockman G.D. (1992). The autolytic (‘suicidase’) system of Enterococcus hirae: From lysine depletion autolysis to biochemical and molecular studies of the two muramidases of *Enterococcus hirae* ATCC 9790. FEMS Microbiol. Lett..

[41] Joris B., Englebert S., Chu C.P., Kariyama R., Daneo-Moore L., Shockman G.D., Ghuysen J.M. (1992). Modular design of the Enterococcus hirae muramidase-2 and Streptococcus faecalis autolysin. FEMS Microbiol. Lett..

[42] Massidda O., Kariyama R., Daneo-Moore L., Shockman G.D. (1996). Evidence that the PBP 5 synthesis repressor (psr) of *Enterococcus hirae* is also involved in the regulation of cell wall composition and other cell wall-related properties. J. Bacteriol..

[43] López R., García E. (2004). Recent trends on the molecular biology of pneumococcal capsules, lytic enzymes, and bacteriophage. FEMS Microbiol. Rev..

[44] Bustamante N., Campillo N.E., García E., Gallego C., Pera B., Diakun G.P., Sáiz J.L., García P., Díaz J.F., Menéndez M. (2010). Cpl-7, a lysozyme encoded by a pneumococcal bacteriophage with a novel cell wall-binding motif. J. Biol. Chem..

[45] García P., García J.L., García E., Sánchez-Puelles J.M., López R. (1990). Modular organization of the lytic enzymes of *Streptococcus pneumoniae* and its bacteriophages. Gene.

[46] Oliveira H., Thiagarajan V., Walmagh M., Sillankorva S., Lavigne R., Neves-Petersen M.T., Kluskens L.D., Azeredo J. (2014). A thermostable Salmonella phage endolysin, Lys68, with broad bactericidal properties against Gram-negative pathogens in presence of weak acids. PLoS ONE.

[47] Perez S., Tvaroška I. (2014). Carbohydrate–protein interactions: Molecular modeling insights. Adv. Carbohydr. Chem. Biochem..

[48] Henrissat B. (1999). Classification of chitinases modules. EXS.

[49] Moreira L.R.S., Filho E.X.F. (2016). Insights into the mechanism of enzymatic hydrolysis of xylan. Appl. Microbiol. Biotechnol..

[50] Li S., Yang X., Bao M., Wu Y., Yu W., Han F. (2015). Family 13 carbohydrate-binding module of alginate lyase from *Agarivorans* sp. L11 enhances its catalytic efficiency and thermostability, and alters its substrate preference and product distribution. FEMS Microbiol. Lett..

[51] Masschalck B., Michiels C.W. (2003). Antimicrobial properties of lysozyme in relation to foodborne vegetative bacteria. Crit. Rev. Microbiol..

[52] Derde M., Guérin-Dubiard C., Lechevalier V., Cochet M.F., Jan S., Baron F., Gautier M., Vié V., Nau F. (2014). Dry-heating of lysozyme increases its activity against *Escherichia coli* membranes. J. Agric. Food Chem..

[53] Wei Z., Wu S., Xia J., Shao P., Sun P., Xiang N. (2021). Enhanced antibacterial activity of hen egg-white lysozyme against *Staphylococcus aureus* and *Escherichia coli* due to protein fibrillation. Biomacromolecules.

[54] Kummer N., Wu T., De France K.J., Zuber F., Ren Q., Fischer P., Campioni S., Nyström G. (2021). Self-Assembly Pathways and Antimicrobial Properties of Lysozyme in Different Aggregation States. Biomacromolecules.

[55] Derde M., Lechevalier V., Guérin-Dubiard C., Cochet M.F., Jan S., Baron F., Gautier M., Vié V., Nau F. (2013). Hen egg white lysozyme permeabilizes *Escherichia coli* outer and inner membranes. J. Agric. Food Chem..

[56] Pellegrini A., Thomas U., Bramaz N., Klauser S., Hunziker P., von Fellenberg R. (1997). Identification and isolation of a bactericidal domain in chicken egg white lysozyme. J. Appl. Microbiol..

[57] Düring K., Porsch P., Mahn A., Brinkmann O., Gieffers W. (1999). The non-enzymatic microbicidal activity of lysozymes. FEBS Lett..

[58] Thammasirirak S., Pukcothanung Y., Preecharram S., Daduang S., Patramanon R., Fukamizo T., Araki T. (2010). Antimicrobial peptides derived from goose egg white lysozyme. Comp. Biochem. Physiol. Part C Toxicol. Pharmacol..

[59] Zavalova L.L., Yudina T.G., Artamonova I.I., Baskova I.P. (2006). Antibacterial non-glycosidase activity of invertebrate destabilase-lysozyme and of its helical amphipathic peptides. Chemotherapy.

[60] Ibrahim H.R., Thomas U., Pellegrini A. (2001). A helix-loop-helix peptide at the upper lip of the active site cleft of lysozyme confers potent antimicrobial activity with membrane permeabilization action. J. Biol. Chem..

[61] Drin G., Antonny B. (2010). Amphipathic helices and membrane curvature. FEBS Lett..

[62] Zschornig O., Paasche G., Thieme C., Korb N., Fahrwald A., Arnold K. (2000). Association of lysozyme with phospholipid vesicles is accompanied by membrane surface dehydration. Gen. Physiol. Biophys..

[63] Giuliani A., Pirri G., Nicoletto S. (2007). Antimicrobial peptides: An overview of a promising class of therapeutics. Open Life Sci..

[64] Dimroth P., Kaim G., Matthey U. (2000). Crucial role of the membrane potential for ATP synthesis by F (1) F (o) ATP synthases. J. Exp. Biol..

[65] Wen S., Yao D., Liu X., Wang F. (2019). A novel fluorescence resonance energy transfer-based high-throughput screening method for generation of lysozyme with improved antimicrobial activity against *Escherichia coli* strains. J. Agric. Food Chem..

[66] Ray B., Bhunia A.K. (2013). Fundamental Food Microbiology.

[67] Carpenter T.S., Parkin J., Khalid S. (2016). The free energy of small solute permeation through the *Escherichia coli* outer membrane has a distinctly asymmetric profile. J. Phys. Chem. Lett..

[68] Nikaido H. (2003). Molecular basis of bacterial outer membrane permeability revisited. Microbiol. Mol. Biol. Rev..

[69] Papo N., Shai Y. (2005). A molecular mechanism for lipopolysaccharide protection of Gram-negative bacteria from antimicrobial peptides. J. Biol. Chem..

[70] Rice A., Wereszczynski J. (2018). Atomistic scale effects of lipopolysaccharide modifications on bacterial outer membrane defenses. Biophys. J..

[71] Brandenburg K., Koch M.H., Seydel U. (1998). Biophysical characterisation of lysozyme binding to LPS Re and lipid A. Eur. J. Biochem..

[72] Ragland S.A., Schaub R.E., Hackett K.T., Dillard J.P., Criss A.K. (2017). Two lytic transglycosylases in *Neisseria gonorrhoeae* impart resistance to killing by lysozyme and human neutrophils. Cell. Microbiol..

[73] Vollmer W., Tomasz A. (2000). The *pgdA* gene encodes for a peptidoglycanN-acetylglucosamine deacetylase in *Streptococcus pneumoniae*. J. Biol. Chem..

[74] Davis K.M., Weiser J.N. (2011). Modifications to the peptidoglycan backbone help bacteria to establish infection. Infect. Immun..

[75] Vollmer W., Tomasz A. (2002). Peptidoglycan N-acetylglucosamine deacetylase, a putative virulence factor in *Streptococcus pneumoniae*. Infect. Immun..

[76] Wang G., Maier S.E., Lo L.F., Maier G., Dosi S., Maier R.J. (2010). Peptidoglycan deacetylation in *Helicobacter pylori* contributes to bacterial survival by mitigating host immune responses. Infect. Immun..

[77] Kaoukab-Raji A., Biskri L., Bernardini M.L., Allaoui A. (2012). Characterization of SfPgdA, a *Shigella flexneri* peptidoglycan deacetylase required for bacterial persistence within polymorphonuclear neutrophils. Microbes Infect..

[78] Melnyk J.E., Mohanan V., Schaefer A.K., Hou C.W., Grimes C.L. (2015). Peptidoglycan modifications tune the stability and function of the innate immune receptor Nod2. J. Am. Chem. Soc..

[79] Moynihan P.J., Clarke A.J. (2011). *O*-Acetylated peptidoglycan: Controlling the activity of bacterial autolysins and lytic enzymes of innate immune systems. Int. J. Biochem. Cell Biol..

[80] Pushkaran A.C., Nataraj N., Nair N., Götz F., Biswas R., Mohan C.G. (2015). Understanding the structure–function relationship of lysozyme resistance in *Staphylococcus aureus* by peptidoglycan *O*-acetylation using molecular docking, dynamics, and lysis assay. J. Chem. Inf. Model..

[81] Shimada T., Park B.G., Wolf A.J., Brikos C., Goodridge H.S., Becker C.A., Reyes C.N., Miao E.A., Aderem A., Götz F. (2010). *Staphylococcus aureus* evades lysozyme-based peptidoglycan digestion that links phagocytosis, inflammasome activation, and IL-1β secretion. Cell Host Microbe.

[82] Laaberki M.H., Pfeffer J., Clarke A.J., Dworkin J. (2011). *O*-Acetylation of peptidoglycan is required for proper cell separation and S-layer anchoring in *Bacillus anthracis*. J. Biol. Chem..

[83] Dillard J.P., Hackett K.T. (2005). Mutations affecting peptidoglycan acetylation in *Neisseria gonorrhoeae* and *Neisseria meningitidis*. Infect. Immun..

[84] Raymond J.B., Mahapatra S., Crick D.C., Pavelka M.S. (2005). Identification of the *namH* gene, encoding the hydroxylase responsible for the *N*-glycolylation of the mycobacterial peptidoglycan. J. Biol. Chem..

[85] Bera A., Biswas R., Herbert S., Kulauzovic E., Weidenmaier C., Peschel A., Götz F. (2007). Influence of wall teichoic acid on lysozyme resistance in *Staphylococcus aureus*. J. Bacteriol..

[86] Fernie-King B.A., Seilly D.J., Davies A., Lachmann P.J. (2002). Streptococcal inhibitor of complement inhibits two additional components of the mucosal innate immune system: Secretory leukocyte proteinase inhibitor and lysozyme. Infect. Immun..

[87] Callewaert L., Van Herreweghe J.M., Vanderkelen L., Leysen S., Voet A., Michiels C.W. (2012). Guards of the great wall: Bacterial lysozyme inhibitors. Trends Microbiol..

[88] Dostal S.M., Fang Y., Guerrette J.C., Scanlon T.C., Griswold K.E. (2015). Genetically enhanced lysozyme evades a pathogen derived inhibitory protein. ACS Chem. Biol..

[89] Peschel A., Vuong C., Otto M., Götz F. (2000). The D-alanine residues of *Staphylococcus aureus* teichoic acids alter the susceptibility to vancomycin and the activity of autolytic enzymes. Antimicrob. Agents Chemother..

[90] Masschalck B., Deckers D., Michiels C.W. (2003). Sensitization of outer-membrane mutants of *Salmonella typhimurium* and *Pseudomonas aeruginosa* to antimicrobial peptides under high pressure. J. Food Prot..

[91] Bader M.W., Navarre W.W., Shiau W., Nikaido H., Frye J.G., McClelland M., Fang F.C., Miller S.I. (2003). Regulation of *Salmonella typhimurium* virulence gene expression by cationic antimicrobial peptides. Mol. Microbiol..

[92] Napier B.A., Burd E.M., Satola S.W., Cagle S.M., Ray S.M., McGann P., Pohl J., Lesho E.P., Weiss D.S. (2013). Clinical use of colistin induces cross-resistance to host antimicrobials in *Acinetobacter baumannii*. mBio.

[93] Niu L.Y., Jiang S.T., Pan L.J., Zhai Y.S. (2011). Characteristics and functional properties of wheat germ protein glycated with saccharides through Maillard reaction. Int. J. Food Sci. Technol..

[94] Aminlari M., Ramezani R., Jadidi F. (2005). Effect of Maillard-based conjugation with dextran on the functional properties of lysozyme and casein. J. Sci. Food Agric..

[95] Alahdad Z., Ramezani R., Aminlari M., Majzoobi M. (2009). Preparation and Properties of Dextran Sulfate—Lysozyme Conjugate. J. Agric. Food Chem..

[96] Corzo-Martínez M., Moreno J.F., Villamiel M., Harte F.M. (2010). Characterization and improvement of rheological properties of sodium caseinate glycated with galactose, lactose and dextran. Food Hydrocoll..

[97] Seo S., Karboune S., L’Hocine L., Yaylayan V. (2013). Characterization of glycated lysozyme with galactose, galactooligosaccharides and galactan: Effect of glycation on structural and functional properties of conjugates. LWT-Food Sci. Technol..

[98] Lesnierowski G., Kijowski J. (2007). Lysozyme. Bioactive Egg Compounds.

[99] Liu S.T., Sugimoto T., Azakami H., Kato A. (2000). Lipophilization of lysozyme by short and middle chain fatty acids. J. Agric. Food Chem..

[100] Liu S., Azakami H., Kato A. (2000). Improvement in the yield of lipophilized lysozyme by the combination with Maillard-type glycosylation. Nahrung.

[101] Syngai G.G., Ahmed G. (2019). Lysozyme: A natural antimicrobial enzyme of interest in food applications. Enzymes in Food Biotechnology.

[102] Abdou A.M., Kim M., Sato K. (2013). Functional proteins and peptides of hen’s egg origin. Bioactive Food Peptides in Health and Disease.

[103] Dickinson E. (2003). Hydrocolloids at interfaces and the influence on the properties of dispersed systems. Food Hydrocoll..

[104] Aminlari L., Hashemi M.M., Aminlari M. (2014). Modified lysozymes as novel broad spectrum natural antimicrobial agents in foods. J. Food Sci..

[105] Amiri S., Ramezani R., Aminlari M. (2008). Antibacterial activity of dextran-conjugated lysozyme against Escherichia coli and Staphylococcus aureus in cheese curd. J. Food Prot..

[106] Wu T., Jiang Q., Wu D., Hu Y., Chen S., Ding T., Ye X., Liu D., Chen J. (2019). What is new in lysozyme research and its application in food industry? A review. Food Chem..

[107] Ferraboschi P., Ciceri S., Grisenti P. (2021). Applications of lysozyme, an innate immune defense factor, as an alternative antibiotic. J. Antibiot..

[108] Ibrahim H.R., Kato A., Kobayashi K. (1991). Antimicrobial effects of lysozyme against Gram-negative bacteria due to covalent binding of palmitic acid. J. Agric. Food Chem..

[109] Nakamura S., Kato A., Kobayashi K. (1991). New antimicrobial characteristics of lysozyme-dextran conjugate. J. Agric. Food Chem..

[110] Nakamura S., Kato A. (2000). Multi-functional biopolymer prepared by covalent attachment of galactomannan to egg-white proteins through naturally occurring Maillard reaction. Nahrung.

[111] Song Y., Babiker E.E., Usui M., Saito A., Kato A. (2002). Emulsifying properties and bactericidal action of chitosan–lysozyme conjugates. Int. Food Res. J..

[112] Park S.I., Daeschel M., Zhao Y. (2004). Functional properties of antimicrobial lysozyme-chitosan composite films. J. Food Sci..

[113] Gaare M., Hussain S.A., Mishra S.K., Ram C. (2013). Natural antimicrobials for preservation of food. Dairy and Food Processing Industry Recent Trends-Part I.

[114] Cegielska-Radziejewska R., Lesnierowski G., Kijowski J. (2008). Properties and application of egg white lysozyme and its modified preparations—A review. Pol. J. Food Nutr. Sci..

[115] Mangalassary S., Han I., Rieck J., Acton J., Dawson P. (2008). Effect of combining nisin and/or lysozyme with in-package pasteurization for control of Listeria monocytogenes in ready-to-eat turkey bologna during refrigerated storage. Food Microbiol..

[116] Stocco G., Cipolat-Gotet C., Cecchinato A., Calamari L., Bittante G. (2015). Milk skimming, heating, acidification, lysozyme, and rennet affect the pattern, repeatability, and predictability of milk coagulation properties and of curd-firming model parameters: A case study of Grana Padano. J. Dairy Sci..

[117] Silvetti T., Brasca M., Lodi R., Vanoni L., Chiolerio F., de Groot M., Bravi A. (2010). Effects of lysozyme on the microbiological stability and organoleptic properties of unpasteurized beer. J. Inst. Brew..

[118] Leone S., Pica A., Merlino A., Sannino F., Temussi P.A., Picone D. (2016). Sweeter and stronger: Enhancing sweetness and stability of the single chain monellin MNEI through molecular design. Sci. Rep..

[119] Wei H., Wang Z., Yang L., Tian S., Hou C., Lu Y. (2010). Lysozyme-stabilized gold fluorescent cluster: Synthesis and application as Hg^2+^ sensor. Analyst.

[120] Becerril R., Gómez-Lus R., Goñi P., López P., Nerín C. (2007). Combination of analytical and microbiological techniques to study the antimicrobial activity of a new active food packaging containing cinnamon or oregano against *E. coli* and *S. aureus*. Anal. Bioanal. Chem..

[121] Pramanik U., Kongasseri A.A., Shekhar S., Mathew A., Yadav R., Mukherjee S. (2021). Structural Compactness in Hen Egg White Lysozyme Induced by Bisphenol S: A Spectroscopic and Molecular Dynamics Simulation Approach. ChemPhysChem.

[122] Jebali A., Hekmatimoghaddam S., Behzadi A., Rezapor I., Mohammadi B.H., Jasemizad T., Yasini S.A., Javadzadeh M., Amiri A., Soltani M. (2013). Antimicrobial activity of nanocellulose conjugated with allicin and lysozyme. Cellulose.

[123] Malhotra B., Keshwani A., Kharkwal H. (2015). Antimicrobial food packaging: Potential and pitfalls. Front. Microbiol..

[124] Rollini M., Nielsen T., Musatti A., Limbo S., Piergiovanni L., Munoz P.H., Gavara R. (2016). Antimicrobial performance of two different packaging materials on the microbiological quality of fresh salmon. Coatings.

[125] Lucera A., Costa C., Conte A., Del Nobile M.A. (2012). Food applications of natural antimicrobial compounds. Front. Microbiol..

[126] Li H., Pan Y., Li C., Yang Z., Rao J., Chen B. (2022). Design, synthesis and characterization of lysozyme–gentisic acid dual-functional conjugates with antibacterial/antioxidant activities. Food Chem..

[127] Niu X., Zhu L., Xi L., Guo L., Wang H. (2020). An antimicrobial agent prepared by N-succinyl chitosan immobilized lysozyme and its application in strawberry preservation. Food Control.

